# New suture materials for midline laparotomy closure: an experimental study

**DOI:** 10.1186/1471-2482-14-70

**Published:** 2014-09-17

**Authors:** Juan M Bellón, Paloma Pérez-López, Raquel Simón-Allue, Sandra Sotomayor, Bárbara Pérez-Köhler, Estefanía Peña, Gemma Pascual, Begoña Calvo

**Affiliations:** 1 Departments of Surgery, Medical and Social Sciences, Faculty of Medicine and Health Sciences, University of Alcalá, Alcalá de Henares, Madrid, Spain; 2 Department of Medicine and Medical Specialities, Faculty of Medicine and Health Sciences, University of Alcalá, Alcalá de Henares, Madrid, Spain; 3 Networking Research Centre on Bioengineering, Biomaterials and Nanomedicine (CIBER-BBN), Madrid, Spain; 4 Aragón Institute of Engineering Research (I3A), University of Zaragoza, Zaragoza, Spain

**Keywords:** Polypropylene, Polyurethane, Polydioxanone, Abdominal wall closure, Midline closure, Laparotomy closure, Barbed sutures, Elastic sutures

## Abstract

**Background:**

Midline laparotomy closure carries a significant risk of incisional hernia. This study examines the behavior of two new suture materials, an elastic material, polyurethane (PUe), and a barbed polydioxanone (PDXb) suture thread in a rabbit model of midline incision closure.

**Methods:**

Three 2-cm midline incisions were made in 68 New Zealand White rabbits. The incisions were closed by running suture using four 3/0 threads: polypropylene (PP) (Surgipro®, *Covidien*), PUe (Assuplus®, *Assut Europe*), PDX (Assufil®, *Assut Europe*) or PDXb (Filbloc®, *Assut Europe*). Animals in each suture group were euthanized 3 weeks and 6 months after surgery. Histological sections of the tissue-embedded sutures were subjected to morphological, collagen expression, macrophage response and uniaxial tensiometry studies.

**Results:**

No signs of wound dehiscence or complications were observed. At 3 weeks, all sutures were surrounded by connective tissue composed mainly of collagen III. PUe showed greater collagen I expression than the other sutures. All sutures elicited a macrophage response that diminished from 3 weeks to 6 months (p < 0.001). This response was similar for the non-reabsorbable sutures (PP and PUe) yet PDXb showed a significantly greater response than the other reabsorbable suture (PDX) at 3 weeks (p < 0.01). At this early time point, the tensile strength of PUe was similar to that of control intact tissue (p > 0.05).

**Conclusion:**

Three weeks after surgery, PUe revealed more collagen I deposition than the remaining materials and this translated to a similar biomechanical behavior to linea alba, that could avoid the appearance of short term dehiscences and thus reduce the incidence of incisional hernia. PDXb provides no additional advantages in their behavior regarding PDX suture.

## Background

Although the use of laparoscopic procedures for abdominal surgery is on the rise, in routine clinical practice, open surgery involving a laparotomy is still often required. Despite several benefits of a transverse laparotomy (e.g., less pain, improved respiratory tolerance) [1-3], the midline laparotomy incision is still the most common approach for many digestive, vascular (especially aortic) and abdominal trauma surgery procedures. This type of laparotomy can be performed quickly and can be extended proximally or distally according to requirements to provide a wide surgical field. On the downside, it is more exsanguinous than the transverse approach.

A midline laparotomy requires opening of the linea alba, which is a weak, tendinous zone of the abdominal wall where fibers of the muscle fascia on each side of the linea alba intersect. The weakness of the linea alba is enhanced when its fibers are vertically sectioned to access the peritoneal cavity. Thus, when repairing or closing the linea alba using sutures, these are subjected to the tension induced by the mechanical forces that act upon it. These forces are that produced by the intraabdominal pressure and the force of the muscle complex comprised of the lateral abdominal muscles whose facias converge at the line alba that tends to separate the edges of the surgical incision. This mechanical factor [4] along with several biological factors [5] is responsible for the high incidence of postoperative incisional hernia, cited as affecting 16 to 20% of cases [6].

The two suture materials most commonly used to close a midline incision are polypropylene and polydioxanone. The former is highly biocompatible and non reabsorbable, while polydioxanone is a reabsorbable polymer material that persists in the mid/long term (for around 180-230 days). Several new suture types have recently been developed including non-reabsorbable and reabsorbable materials with elastic properties. These elastic materials have been tested in multicenter clinical trials [7] though follow up times have been insufficiently long for conclusive results to be drawn regarding their behavior. Another type of suture that has recently appeared on the market [8], the barbed suture, is not really new. It was patented back in 1964 [9] and has been reintroduced for several surgical applications such as plastic surgery [10] and digestive surgery [11,12]. Barbed sutures have also been experimentally tested for the closure of fascias [13] and tendons [14].

This study was designed to test two new suture materials not previously assessed, a non-reabsorbable material with elastic properties and a reabsorbable barbed suture thread. Both these materials are similar to those existing on the market. Our main objective was to determine whether these new sutures offer any benefits over conventional suture materials in terms of their biological and mechanical behavior when used to close a midline laparotomy. For this purpose, we used a model in New Zealand white rabbit previously established and validated by our group for abdominal wall repair studies.

## Methods

### Experimental animals

Sixty eight New Zealand white rabbits of mean weight 3000 g were housed and handled during the entire study period in accordance with the recommendations of the Guide for the Care and Use of Laboratory Animals of the National and European Institutes of Health (Spanish law 32/2007, Spanish Royal Decree 1201/2005, European Directive 2010/63/UE and European Convention of the Council of Europe ETS123). All procedures were performed at the Animal Research Centre, Alcalá University. The study protocol received institutional review board approval by the Committee on the Ethics of Animal Experiments of the University of Alcalá (registered code: ES280050001165). All animals were housed in individual cages and were allowed free access to water and a maintenance diet (PANLAB®) in a 12-hour light/dark cycle, with room temperature at 21 ± 2°C.

The material and methods have been adhered to the ARRIVE guidelines Kikenny, Plos one 2010, for the publication of animal studies.

To minimize pain, the animals were administered 0.05 mg/kg buprenorphine (Buprecare®, DivasaFarmavic) 1 hour before and 3 days after the surgical procedure. Anesthesia was induced with a mixture of ketamine hydrochloride (Ketolar®, Parke-Davis) (70 mg/Kg), diazepam (Valium®, Roche)(1.5 mg/Kg) and chlorpromazine (Largactil®, Rhone-Poulenc) (1.5 mg/Kg) administered intramuscularly.

### Suture materials

**Figure 1:**
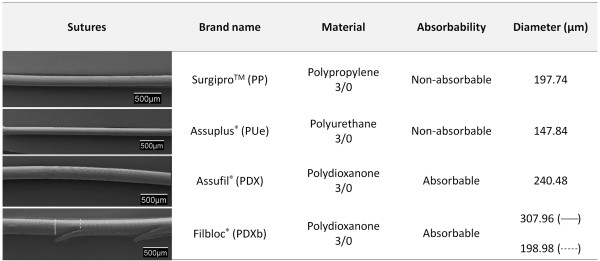
**Characteristics of the different sutures used in the study.** Table showing the brand name, material composition, suture absorbability and diameter of the suture. (SEM images; 20x).

The suture materials tested were (Figure 1):

– Surgipro® 3/0 (PP) (Covidien, USA): a non-reabsorbable polypropylene monofilament.

– Assuplus® 3/0 (PUe) (Assut Europe, Italy): a non-reabsorbable elastic polyurethane monofilament.

– Assufil® 3/0 (PDX) (Assut Europe, Italy): a reabsorbable polydioxanone monofilament.

– Filbloc® 3/0 (PDXb) (Assut Europe, Italy): a reabsorbable unidirectional barbed polydioxanone monofilament.

### Surgical technique

**Figure 2:**
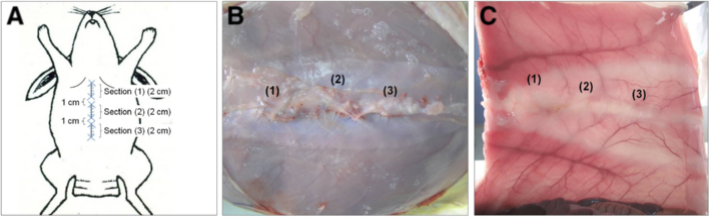
**Experimental design and macroscopic aspect of the laparotomies after euthanasia.** Diagram of the experimental design showing the 3 incisions made along the linea alba. Each incision was 2 cm in length and placed in the upper zone (1), middle zone (2) and lower zone (3) of the midline with 1 cm vertical separation between each one **(A)**. Macroscopic images of the sutures 3 weeks **(B)** and 6 months **(C)** after closure. All the sutures appear well localized in the three zones of the linea alba. Subcutaneous **(B)** and peritoneal sides observed by transillumination of the linea alba **(C)**.

Using a sterile surgical technique, each animal was subjected to 3 laparotomies 2 cm in length along the linea alba in the upper zone (1), middle zone (2) and lower zone (3) with a vertical separation between each incision of 1 cm (Figure 2A). The first incision was made 3 cm from the xiphoid process to ensure that all the sutures were placed at the same level in each animal. This laparotomy configuration with 1-cm interuptions was designed to obtain samples for accurate biomechanical tests. These tests could therfore be performed on complete sutures. The incisions were closed by running suture in a single plane that comprised the margins of the fascia along with the peritoneum. All closures were performed by the same surgeon (JMB) using a 4:1 suture length:wound length ratio [15] and the small stitches technique [16].

During the study course, the animals were inspected daily for signs of wound dehiscence, seroma or surgical wound infection.

### Experimental design

In each animal, the three incisions made were closed using one of the four suture materials to establish the following study groups:

– Group I (control, n = 12): animals not undergoing surgery used as controls (6 for each time point)

– Group II (n = 14): animals undergoing three incisions closed using PP

– Group III (n = 14): animals undergoing three incisions closed using PUe

– Group IV (n = 14): animals undergoing three incisions closed using PDX

– Group V (n = 14): animals undergoing three incisions closed using PDXb.

In the experimental groups (II-V), 7 animals were sacrificed following protocols for experimental animal euthanasia in a CO2 chamber at the time points 3 weeks and 6 months after surgery. Of these 7 animals, 4 were used for the biomechanical tests and 3 for the histological studies.

### Morphological studies

Suture samples for Scanning electron microscopy (SEM) observation were metalized with gold palladium and examined under a Zeiss scanning electron microscope (DSM-950) (Carld Zeiss, Oberkochen, Germany).

For light microscopy, control and suture/tissue samples were fixed in F13 solution (60% ethanol, 20% methanol, 7% polyethylene glycol, 13% distilled water), embedded in paraffin, sliced into 5 μm sections, and stained with Masson’s trichrome (Goldner–Gabe) stains.

### Collagen expression

Collagen was detected in the control and suture/tissue samples by immunofluorescent labeling. Samples of tissue from each of the animals included in the study were fixed in F13 solution, embedded in paraffin and cut into 5 μm thick sections. Once cut, the sections were deparaffinated, hydrated and equilibrated in Phosphate buffered saline (PBS). Non-specific protein interactions were blocked using 3% BSA (bovine serum albumin) and specimens incubated with the monoclonal antibodies anti-collagen I (1:400 in PBS) (Sigma, St. Louis, MO, USA) and anti-collagen III (1:500 in PBS) (Medicorp, Montreal, Canada). A secondary antibody conjugated to rhodamine was added to the incubation mixture. Negative controls were incubated with 3% BSA instead of the primary antibody. Cell nuclei were counterstained with 4′, 6′-diamino-2-phenylindole (DAPI). The stained sections were examined under a confocal microscope Leica SP5 (Leica Microsystems, Wetzlar, Germany) to detect fluorescence. This work was carried out at the confocal microscopy facility of the University of Alcala de Henares (UAH) and CIBER-BBN located at the Cell Culture Unit: http://www3.uah.es/caimedicinabiologia/index.php/cultivos-celulares.

### Macrophage response

Paraffin-embedded tissues were immunolabelled using a monoclonal antibody against rabbit macrophages RAM-11 (DAKO M-633, USA) in the alkaline phosphatase-labeled avidin-biotin procedure. The method consists of the following steps: blockade with 3% BSA, incubation with the primary antibody (1:50 in PBS), incubation with immunoglobulin G (IgG) and biotin, and labeling with avidin. These steps were conducted at room temperature. The images were developed using a chromogenic substrate containing naphthol phosphate and fast red. Negative controls were incubated with 3% BSA instead of the primary antibody. Nuclei were contrasted for 5 min with acid hematoxylin. Labeled macrophages were quantified by performing counts in 10 microscopy fields (magnification x20) per sample under a Zeiss light microscope (Carl Zeiss, Oberkochen, Germany). Results are expressed as mean percentages of labeled cells out of the total number of cell nuclei per section.

### Biomechanical study

For the mechanical tests, three control and three suture/tissue samples for each laparotomy of a width/length ratio around 1/7 were cut from each rabbit to fulfil the criterion for uniaxial tension. Sample length, width and thickness were measured using a digital calliper.

Uniaxial tensile tests were performed under displacement control on an INSTRON 3340 microtester with a 1 (kN)-fullscale load cell. To maintain quasi-static testing conditions, the applied displacement rate was 5 mm/min. Load and displacement until complete sample rupture were used to calculate the stretch of the sample as λ = (L0 + ∆L)/L0, where L0 is the starting distance between the clamps, ∆L is the clamp displacement and σ = N/CSA λ is the Cauchy stress factor in the direction of stretch, where N is the applied load and CSA the initial section of the sample.

Thus, the results were expressed as the Cauchy values stress (in MPa) supported by three different levels of strain, or stretch (λ), and represent means for the three incisions made in each animal.

### Statistical analysis

Macrophage percentages and tensile strengths are provided as means ± SEM and compared using the Mann Whitney *U*-test and the Wilcoxon test. All statistical analyses were performed using the GraphPad Prism 5 computer package for Windows. Significance was set at p < 0.05.

## Results

No signs of infection, seroma, wound dehiscence and/or other complications were detected in any of the animals. When suture/tissue samples were collected at 3 weeks postsurgery, all sutures were clearly visible in the three established zones along the linea alba (Figure 2B).At the 6-month time point, the PP and PUe sutures could still be seen in the 3 zones, while the surgical wounds incorporating the reabsorbable PDX and PDXb sutures showed the same macroscopic appearance as the control intact linea alba. However, under transillumination of the linea alba, the suture zones could be well distinguished as zones of contrast produced by the scar tissue (Figure 2C).

### Morphological study

**Figure 3:**
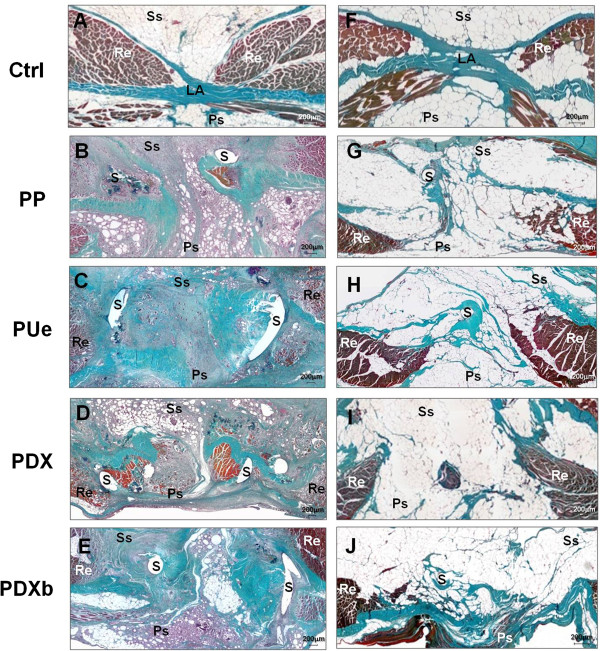
**Light microscopy images.** Panoramic view of the control linea alba at 3 weeks **(A)** and 6 months **(F)** and in the midline closure performed using the different sutures at 3 weeks **(B-E)** and 6 months **(G-J)**. (Masson’s trichrome stain, 100x). s: suture, LA: linea alba, Re: rectus abdominis muscles, Ss: subcutaneous side, Ps: peritoneal side, PP: Surgipro™, PUe: Assuplus®, PDX: Assufil® and PDXb: Filbloc®.

In control tissue samples, the linea alba could be distinguished by Masson’s trichrome staining and light microscopy observation as composed of connective tissue arising from the lateral zones of the premuscular and retromuscular fascia. On both sides of the linea alba, the fibers of the abdominal rectus muscles could be seen. Both the subcutaneous and peritoneal sides showed an abundance of adipose tissue (Figure 3A, F).In the suture groups, the zone corresponding to the linea alba had thickened at 3 weeks postsurgery and there was an abundance of disorganized connective tissue, which was highly cellular in the PUe and PDXb suture groups (Figure 3B-E).At 6 months, all suture/tissue samples showed the significant development of adipose tissue in zones close to the suture. In the PP and PUe closures, a compact connective tissue was observed around the suture filaments (Figure 3G-J).

### Collagen expression

#### *Collagen type III*

**Figure 4:**
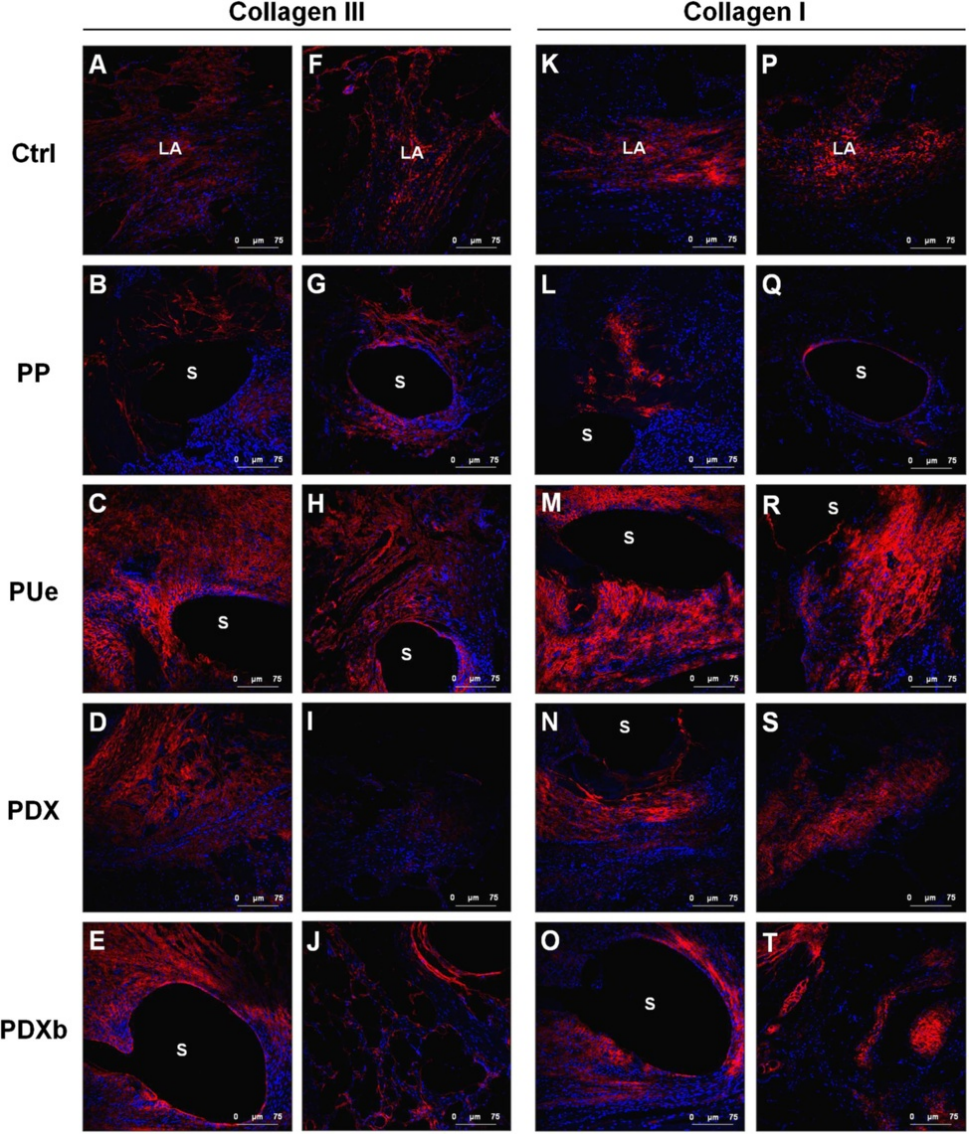
**Expression and localization of collagen type III and I.** Immunofluorescence detection of immature collagen III in the control linea alba at 3 weeks **(A)** and 6 months **(F)** and in the midline closure performed using the different sutures at 3 weeks **(B-E)** and 6 months **(G-J)**. Collagen fibers appear in red and cell nuclei in blue (DAPI stain). Confocal light microscopy (200x). Immunofluorescence labeling of mature collagen type I along the control linea alba at 3 weeks **(K)** and 6 months **(P)** and after closing the laparotomy incisions using the different suture threads at 3 weeks **(L-O)** and 6 months **(Q-T)**. Collagen fibers appear in red and cell nuclei in blue (DAPI stain). Confocal light microscopy (200x). s: suture, LA: linea alba, PP: Surgipro™, PUe: Assuplus®, PDX: Assufil® and PDXb: Filbloc®.

Immunostaining for collagen type III revealed slight expression at the linea alba and the different fascias between muscle layers both at 21 and 180 days (Figure 4A, F).In all the study groups, immature collagen was detected in the tissue around the suture stitches and in the scar tissue in the laparotomy zone at 3 weeks. At this time point, both the non-reabsorbable PUe and absorbable PDXb showed more collagen III expression than the PP and PDX sutures (Figure 4B-E). At 6 months, the collagen III expression level remained stable in the non-reabsorbable PUe and PP sutures yet diminished slightly in the reabsorbable PDX and PDXb sutures (Figure 4G-J).

#### *Collagen type I*

Collagen type I immunostaining indicated that the linea alba and the different fascias between muscle layers were comprised mostly of mature type I collagen at both 21 and 180 days (Figure 4K, P).The labeling intensity observed for mature collagen was greater than for immature collagen. Collagen I was detected around the sutures and in the center of the laparotomy zone. Of all the study groups, the PP sutures showed least collagen I expression at both time points (Figure 4L, Q).No significant differences were found in collagen type I expression between 21 (Figure 4L-O) and 180 days (Figure 4Q-T). In the short and long term, the elastic PUe sutures showed more collagen I expression than the remaining suture types (Figure 4M, R).

### Macrophage response

**Figure 5:**
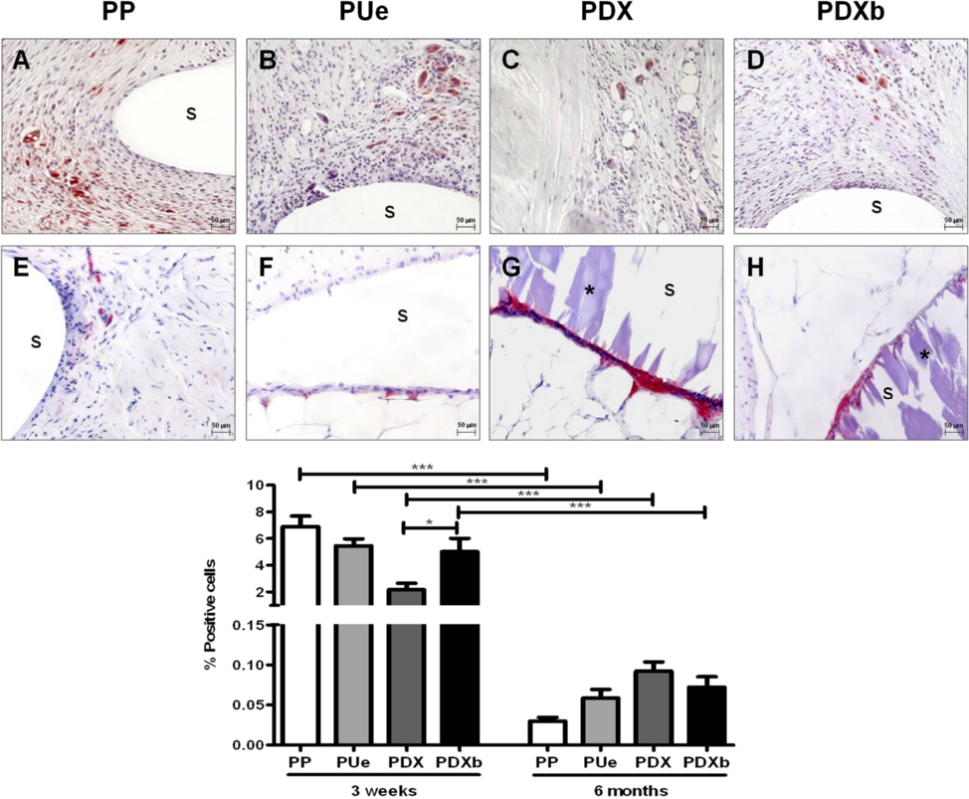
**Macrophage response to suture materials.** Immunodetection of RAM-11 rabbit macrophages at 3 weeks **(A-D)** and 6 months **(E-H)** post intervention (Top panel, 200x). s: suture; *: non degraded remains of PDX. Results expressed as the mean ± SEM of RAM-11 positive macrophage percentages. Mann-Whitney *U* Test. *, p < 0.05; ***, p < 0.001. PP: Surgipro™, PUe: Assuplus®, PDX: Assufil® and PDXb: Filbloc®.

In the short term, active macrophages localized in zones close to the different sutures (Figure 5A-D). At six months, this response ceased and only some giant foreign body reaction cells surrounding the undegraded remains of PDX and PDXb could be observed (Figure 5E-H).

The non-reabsorbable sutures (PP and PUe) showed a similar macrophage response at both time points. However, while this response differed significantly between the two suture materials at 3 weeks, no differences were observed at 6 months.All sutures showed a significant reduction in macrophage labeling from 3 weeks to 6 months (Figure 5).

### Biomechanical study

**Figure 6:**
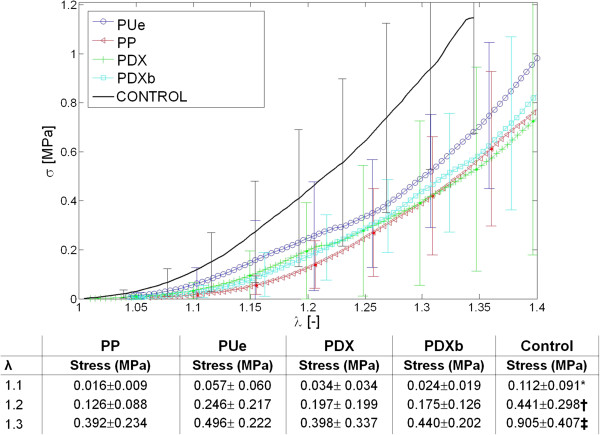
**Cauchy values stress (MPa) at 3 weeks.** The results are for three different strains (λ) applied to the different sutures and healthy control tissue 3 weeks after laparotomy closure. Provided in the table are the means ± SD of three stress values obtained from the tension-deformation curve 3 weeks after laparotomy closure (*, p < 0.05 vs. PP/PDX/PDXb; †, p < 0.05 vs. PP/PDXb; ‡, p < 0.05 vs. all the groups). PP: Surgipro™, PUe: Assuplus®, PDX: Assufil® and PDXb: Filbloc®.

**Figure 7:**
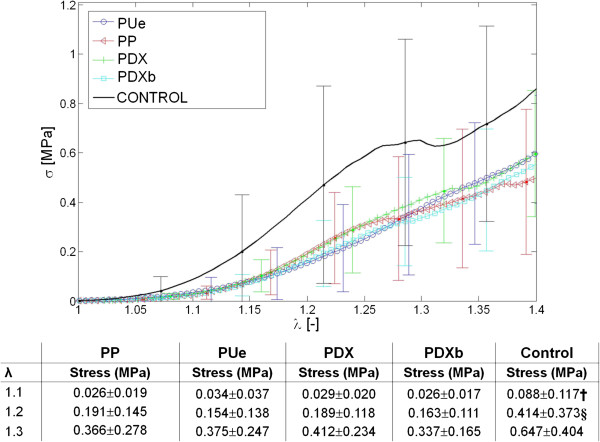
**Cauchy values stress (MPa) at 6 months.** The results (MPa) for three different strains (λ) applied to the different sutures and healthy control tissue 6 months after laparotomy closure. Provided in the table are the means ± SD of three stress values obtained from the tension-deformation curve 6 weeks after laparotomy closure (†, p < 0.05 vs. PP/PDXb; §, p < 0.05 vs. PUe/PDXb). PP: Surgipro™, PUe: Assuplus®, PDX: Assufil® and PDXb: Filbloc®.

The closed linea alba should show a similar mechanical response to the healthy intact abdominal wall, so we compared the mechanical behavior of suture/tissue and healthy tissue samples over time.At 3 weeks, when the different sutures were compared for low levels of strain (λ ≤ 1.2 or 20%) (Figure 6) the behavior observed for PP, PDX and PDXb versus healthy tissue (control) was different. Control tissue showed significant higher stress or tension values (p < 0.05) than the values recorded in the latter three groups. However, PUe showed a similar response to control tissue (p > 0.05). At greater strain levels, (λ ≥ 1.3 or 30%), the four types of sutures behaved similarly and none was able to reproduce the behavior of control healthy tissue (p < 0.05 for sutures vs controls).At 6 months (Figure 7), some differences emerged between the biomechanical response shown by sutures and healthy tissue. For low levels of strain, stress values for control samples varied with respect to PP, PUe and PDXb (p < 0.05), yet no such differences were observed for greater strains. The high variation in the stress values recorded for the samples, however, confers this finding low statistical power.

## Discussion

The most appropriate biomaterials for laparotomy closure able to reduce the incidence of incisional hernia have yet to be discovered [17]. Although, the most important factor determining the failure of the fascia is not the suture type, since many different factors are influencing (individual’s own biological factors, presence of infection, technical skill of the surgeon, etc…), both clinical and experimental studies [18,19] have examined the influence of different suture types on the closure of a midline laparotomy independently of other important factors such running versus interrupted suture, distance between stitches and the width of the tissue margin included in the suture [20].

Thus, important factors for good laparotomy closure are the suture technique used, which in many cases is surgeon dependent [21], and the choice of suture material. The debate about the best suture technique among other aspects has revolved around whether it is best to place a running suture or loose stitches.

Most authors have argued for the use of a running suture [22-26] since it evenly distributes tension along the suture line, lowering the risk of tissue ischemia. The 4:1 suture length: wound length ratio established by Jenkins [15] is well accepted and has been supported by experimental [27] and clinical studies. In the latter, Israelsson et al. [28] reported that a suture:wound length ratio greater than 4 reduced the incidence of incisional hernia. Besides the need to comply with this ratio, other preclinical [16,29] and clinical [30] studies have identified the importance of using small stitches to close the abdominal wall [31].

In the present study, we opted for a running suture placed at a 4:1 suture:wound length ratio using the small stitches technique although we included the peritoneum besides the fascial margins in the suture, sparing the muscular plane. No signs of wound dehiscence were observed in any of the present animals.

According to some authors [25], the ideal suture material should have no impact on the incidence of infection, should avoid patient discomfort and should not induce sinus formation in the surgical wound. In the present study, we tested two new suture materials with elastic properties: PUe, composed of a monofilament of polyurethane polymer, which renders it non reabsorbable; and PDXb made of the already known polydioxanone but of a barbed structure, which makes it self-retaining avoiding the need for knots.

Our main objective was to determine the biological behavior of these new sutures and examine whether this behavior had implications for the final mechanical strength of the suture site in a midline laparotomy. The suture materials compared were both non reabsorbable (PP, PUe) and reabsorbable (PDX, PDXb).

Our histological findings based on immunolabelling for collagens indicated that the PUe suture showed the greater expression of collagen I in the short term (21 days) which was similar to control levels. This behavior of the PUe suture could be related to its elastic properties resulting in less tension generated along the suture line. In an experimental study, Höer et al. [32] observed that a tension-free closure promotes collagen deposition in the repair zone. At 6 months, the expression of this collagen type I remained high in PUe compared to all the other groups.

The macrophage response to the sutures observed here was similar for the two non-reabsorbable threads yet greater at 3 weeks than 6 months for PDXb compared to PDX. This more intense response could be attributed to the greater thickness of barbed PDX than conventional PDX. By 6 months, these differences had vanished. Over time (3 weeks versus 6 months), a significantly reduced macrophage response was observed for all the suture types. This indicates the good biocompatibility of all these materials.

The biomechanical behavior of the sutures was examined based on the results of prior studies performed in human patients by other authors [33]. According to these authors, traction measurements on the linea alba indicate a higher tensile strength along the longitudinal axis than the transverse axis. The forces that act upon a closed linea alba tend to pull apart the sutured edges of the incision. This is due both to effects of intraabdominal pressure and the cylindrical shape of the abdominal cavity. According to the law of Laplace, force vectors distributed transversely across cylinder walls cause the most medial margins of a cylinder to separate [34]. When this model is translated to the abdominal cavity, the anterior medial margin is the midline or linea alba. Our biomechanical tests were thus performed on strips of tissue including the whole suture line and traction was applied to an axis perpendicular or transverse to this line. These tests were designed to reliably determine the behavior of the sutures made along the linea alba. By interrupting the suture in three zones of the linea alba, we were able to include the whole suture line in each test without the need to cut each suture line into sections.

Our findings indicate that the suture made using the new elastic suture material (PUe) showed a behavior that resembled that of the intact linea alba shortly after surgery (3 weeks). The tension-deformation curve of the tissue strip incorporating this material did not differ significantly from the control curve. This finding coincided with the already mentioned collagen type I expression found in these samples providing strength to the repair process. We believe that this mechanical behavior of PUe is important, since in line with the findings of others [35,36], it is within the first month of a laparotomy closure that small dehiscences may form at the suture line and these may later give rise to incisional hernias. Behaving similarly to control tissue, an elastic suture thread could theoretically avoid the appearance of these small dehiscences and thus reduce the incidence of incisional hernia. In a clinical, prospective, clinical study of laparotomy closure [7] in which an elastic suture thread made of a different polymer to PUe was used, a lower incidence of incisional hernia was observed after one year of follow up, though the difference was not significant compared to controls. In a recent experimental study on tendons, elastic suture threads were also reported to show improved mechanical performance [14].

At six months, the four suture materials tested here showed similar biomechanical behavior. As expected, the new tissue generated never reached control levels of stress yet it is this tissue itself that conditions and leads to the similar mechanical behavior of the different materials. These longer-term findings are in agreement with those of studies that have compared polydioxanone versus polypropylene [37] and the use of a barbed polydioxanone suture thread versus conventional polydioxanone for fascial closure [13]. This finding indicates that modifying the structure of a monofilament suture thread such as in the barbed sutures fails to improve tensile strength. Barbed sutures do, however, offer other benefits such as avoiding the need for knots and a good tissue retention capacity that avoids the slippage produced by monofilament sutures. This aspect is interesting; in a comparative study [38] polypropylene was noted to cause more slippage than polydioxanone.

This preclinical study has its limitations, especially related to the biomechanical response of the suture materials. Thus, the biomechanical properties of the materials examined in the rabbit may not be translatable to the situation in humans because of the different intraabdominal pressure that acts upon the anterior wall of the abdomen in humans (due to an upright position and other mechanical requirements). In contrast, the biological behavior of the materials tested is more easily extrapolatable to what might occur when used in clinical practice.

## Conclusions

In conclusion, the findings of this study indicate that: a) at three weeks, the elastic PUe suture showed greatest collagen type I deposition which was correlated with a tensile strength resembling that of the control intact tissue, that could avoid the appearance of short term dehiscences and thus reduce the incidence of incisional hernia; b) the macrophage response diminished significantly from 3 weeks to 6 months for all the suture materials; and c) the barbed PDXb suture showed similar behavior to the PDX suture, providing no additional benefits.

## Abbreviations

PU: Polyurethane; PUe: Elastic polyurethane; PDX: Polydioxanone; PDXb: Barbed polydioxanone; PP: Polypropylene; SEM: Scanning electron microscopy; PBS: Phosphate buffered saline; BSA: Bovine serum albumin; DAPI: 4′, 6′-diamino-2-phenylindole.

## Competing interests

The authors declare that they have no competing interests.

## Authors’ contributions

JMB, GP, EP and BC conceived of the study, participated in its design and coordination and wrote the manuscript. RSA, PPL and SS helped to draft the manuscript. JMB, GP, SS and BC participated in the analysis and interpretation of the results. SS, PPL, BPK, EP and RSA performed the data collection and statistical analysis. All authors read and approved the final manuscript.

## Pre-publication history

The pre-publication history for this paper can be accessed here:

http://www.biomedcentral.com/1471-2482/14/70/prepub
